# Monoclonal Antibodies against SARS-CoV-2: Potential Game-Changer Still Underused

**DOI:** 10.3390/ijerph182111159

**Published:** 2021-10-24

**Authors:** Ivan Gentile, Alberto Enrico Maraolo, Antonio Riccardo Buonomo, Mariano Nobile, Prisco Piscitelli, Alessandro Miani, Nicola Schiano Moriello

**Affiliations:** 1 Section of Infectious Diseases, Department of Clinical Medicine and Surgery, University of Naples Federico II, 80131 Naples, Italy; antonioriccardobuonomo@gmail.com (A.R.B.); mariano.nobile1991@gmail.com (M.N.); s.nicola@runbox.com (N.S.M.); 2 Staff UNESCO Chair on Health Education and Sustainable Development, University of Naples Federico II, 80131 Naples, Italy; priscofreedom@hotmail.com; 3 First Division of Infectious Diseases, Cotugno Hospital, AORN Dei Colli, 80131 Naples, Italy; albertomaraolo@yahoo.com; 4 Euro Mediterranean Scientific Biomedical Institute, 1000 Bruxelles, Belgium; 5 Department of Environmental Science and Policy, University of Milan, 20122 Milan, Italy; Alessandro.miani@gmail.com

**Keywords:** COVID-19, monoclonal antibodies, lethality, hospitalizations, savings

## Abstract

Even several months after the start of a massive vaccination campaign against COVID-19, mortality and hospital admission are still high in many countries. Monoclonal antibodies against SARS-CoV-2 are the ideal complement to vaccination in infected subjects who are at high risk for progression to severe disease. Based on data of the Italian Ministry of Health, in the period April–August 2021, monoclonal antibodies were prescribed to 6322 patients. In the same period, 70,022 patients over 70 years old became infected with SARS-CoV-2. Even considering that all monoclonal antibodies were prescribed to this category of patients, we calculated that only 9% of these subjects received the treatment. Moreover, using efficacy data provided by clinal trials, we estimated the potential benefit in terms of reduction of hospital admissions and deaths. Considering utilisation of monoclonal antibodies in half infected patients over 70 years, we estimated that hospital admissions and deaths might have been reduced by 7666 and 3507, respectively. Finally, we calculated the economic benefit of monoclonal use. In the same scenario (50% use of monoclonal antibodies to patients over 70), we estimated potential savings of USD 117,410,105. In conclusion, monoclonal antibodies were used in a small proportion of patients over 70 in Italy. A more extensive use might have resulted in a marked decrease in hospital admissions, deaths and in conspicuous saving for the health system.

## 1. Introduction

Vaccination is the most effective way to protect from coronavirus disease 2019 (COVID-19), although it cannot represent the only way to cope with the ongoing pandemic [1,2,3,4]. Italy is among the Western European countries most hit by the pandemic [5]. Notwithstanding campaigns to provide extensive vaccine coverage and information about immunisation, part of the population remains sceptical about the vaccination. Several factors, namely safety concerns, doubts about effectiveness, and the speed with which vaccines were developed, contribute to vaccine hesitancy and it is unlikely that everyone will eventually agree to vaccination. Moreover, vaccination is less effective in immunosuppressed subjects such as transplanted patients and those with haematological malignancies. A significant proportion of these subjects will not develop an effective response and will be at risk of a severe disease.

Currently, the only effective therapy in patients with mild COVID-19 and who are risk of progression to severe disease is the early (ideally within 4–5 days from symptoms onset) administration of monoclonal antibodies against the spike protein. These drugs act through a selective binding to the receptor binding domain of the spike protein, therefore inhibiting the interaction between SARS-CoV-2 and the host cell and blocking virus entrance. Monoclonal antibodies for SARS-CoV-2 infection can be administered by IV infusion or SQ injections and, according to recommendations of the Italian Minister of Health, should only be administered in healthcare settings in order to cope with a possible adverse reaction [6,7]. 

At the present time, three monoclonal antibodies treatment are approved for use in Italy: Bamlanivimab-Etesevimab, Casirivimab-Imdevimab, and Sotrovimab [7]. Those therapies showed similar benefits in term of reduction both in hospital admissions and deaths [8,9].

In the attempt to provide real-life data on the current use of monoclonal antibodies against SARS-CoV-2 in western countries, we investigated the use of monoclonal antibodies in Italy and estimated the impact of a more extensive use of this therapy on hospital admissions, deaths, and costs.

## 2. Methods

We determined the number of new SARS-CoV-2 infections, hospitalisations, ICU admissions and death due to coronavirus disease in Italy between 2 April 2021 (the day Italy approved this therapy) and 5 August 2021. Data of new cases of SARS-CoV-2 infection are publicly available on the website of the Italian Ministry of Health [7]. Using WebPlotDigitizer software, we extracted all other data from the graphs on the web page of the Italian National Institute of Nuclear Physics that were provided by the Italian National Institute of Health [8]. We assumed three scenarios of monoclonal antibody use in people over 70 years of age (rates of use of 50, 70, or 90%), and estimated their respective consequences on hospital admissions, deaths, and costs, as if they were administered according to the above-mentioned rates assuming the same efficacy showed data reported in the REGEN-COV trial (NCT04425629) [6]. Similar efficacy data were published about Bamlanivimab plus Etesevimab and Sotrovimab [9,10]. We also estimated the potential economic benefit related to the use of monoclonal antibodies. The price of a monoclonal antibody was estimated at USD 1250 per treatment [11]. The cost per hospitalisation was estimated as follows: without ICU, USD 16,924; with ICU, USD 57,934 [12]. We assumed a ratio between hospitalisation without ICU and with ICU of 9:1, as seen in the target population in the study period, therefore the mean hospitalisation cost was set at USD 21,025.

## 3. Results and Discussion

As shown in Table 1, 777,502 cases of SARS-CoV-2 infection were recorded during the study period. The incidence fluctuated from a high of 110,594 cases per week to a low of 5099 cases per week. A total of 70,022 people were over the age of 70 years. There were 57,740 hospital admissions, of which 21,503 were in people over 70 years of age. A total of 18,422 deaths were recorded, 9963 of which were among people over the age of 70.

In Italy, monoclonal antibodies use is approved for patients with SARS-CoV-2 infection and with at least one risk factor for progression towards a severe disease. Age over 65 years is one of these factors. In the period considered, monoclonal antibodies were prescribed for 6322 patients. It is not known how many monoclonal antibodies were prescribed in patients over 70. However, even assuming that all 6322 treatments were administered in patients in this age group, this figure represents 9.02% of all patients over 70.

Projecting a reduction in hospital admissions and deaths based on the data available (6), we estimate that, during the study period, hospital admissions and deaths would have declined by 13,798 and 6313, respectively, considering a rate of utilisation of 90% as shown in Table 2. Complete projections are reported in Table 2.

Regarding costs, the expenditure for the acquisition of monoclonal antibodies is counterbalanced by the saving of hospital admissions. As a consequence, we estimate total savings of USD 211,338 million dollars in the case of 90% use (see Table 3).

We acknowledge that this study presents several limitations: first of all, we projected the efficacy of monoclonal antibodies estimating similar effectiveness in real life compared to that from clinical trials. For example, the population considered in the present study was significantly different from the one enrolled in the trials, at least in terms of median age; moreover, the estimate of the use of monoclonal antibodies is rough because it does not consider other categories in which monoclonal antibodies are indicated (e.g., subjects with immunodeficiency, diabetes, obese, etc.).

## 4. Conclusions

Use of monoclonal antibodies for SARS-CoV-2 infection between April and August 2021 in Italy has been very low. Even looking at the most prudential estimates, a more extensive use of these therapies could have prevented a high number of hospitalisations and deaths among patients over 70, together with a positive impact on COVID-19 related costs. These data should prompt authorities to create a higher interaction between general practitioners and facilities for monoclonal antibodies infusion, in order to refer patients at high risk of deterioration as soon as possible.

## Figures and Tables

**Table 1:** Data obtained in the period of interest.

	Total	Patients over 70 Years (Rate)
NEW SARS-CoV-2 INFECTIONS	777,502	70,022 (9%)
HOSPITAL ADMISSIONS	57,440	21,503 (37.4%)
DEATHS	18,422	9963 (54%)
MONOCLONAL ANTIBODIES ADMINISTRED	6322	n.a.

**Table 2:** Projected hospital admissions and deaths in subjects above 70 years of age who received monoclonal antibodies.

	50% M.A. USE	70% M.A. USE	90% M.A. USE
HOSPITAL ADMISSIONS	13,837 (15,944–12,590)	10,771 (13,721–9025)	7705 (11,498–5460)
REDUCTIONS	7666 (5559–8913)	10,732 (7782–12,478)	13,798 (10,005–16,043)
REDUCTIONS (%)	35.7% (25.9–41.5%)	49.9% (36.2–58.0%)	64.2% (46.5–74.6%)
DEATHS	6456 (8389–5624)	5053 (7759–3889)	3650 (7130–2153)
REDUCTIONS	3507 (1574–4339)	4910 (2204–6074)	6313 (2883–7810)
REDUCTIONS (%)	35.2% (15.8–43.6%)	49.3% (22.1–61.0%)	63.4% (28.4–78.4%)

M.A.: monoclonal antibodies; in parenthesis data with 95% C.I.

**Table 3:** Economic implications of extensive monoclonal prescription in patients over the age of 70.

PERCENTAGE OF M.A. USE	50%	70%	90%
NUMBER OF A.M. ADMINISTERED	35,011	49,015	63,020
COST OF M.A. (USD)	43,763,750	61,269,250	78,774,750
SAVING FOR H.A. REDUCTION (USD)	161,173,855	225,643,397	290,112,939
NET SAVING (USD)	117,410,105	164,374,147	211,338,189

H.A.: Hospital Admissions; M.A.: monoclonal antibodies.

## Data Availability

The data presented in this study are available on request from the corresponding author.

## References

[1] Sadoff J., Le Gars M., Shukarev G., Heerwegh D., Truyers C., de Groot A.M., Stoop J., Tete S., Van Damme W., Leroux-Roels I. (2021). Interim Results of a Phase 1–2a Trial of Ad26.COV2.S COVID-19 Vaccine. N. Engl. J. Med..

[2] Folegatti P.M., Ewer K.J., Aley P.K., Angus B., Becker S., Belij-Rammerstorfer S., Bellamy D., Bibi S., Bittaye M., Clutterbuck E.A. (2020). Safety and immunogenicity of the ChAdOx1 nCoV-19 vaccine against SARS-CoV-2: A preliminary report of a phase 1/2, single-blind, randomised controlled trial. Lancet.

[3] Baden L.R., El Sahly H.M., Essink B., Kotloff K., Frey S., Novak R., Diemert D., Spector S.A., Rouphael N., Creech C.B. (2021). Efficacy and Safety of the mRNA-1273 SARS-CoV-2 Vaccine. N. Engl. J. Med..

[4] Polack F.P., Thomas S.J., Kitchin N., Absalon J., Gurtman A., Lockhart S., Perez J.L., Marc G.P., Moreira E.D., Zerbini C. (2020). Safety and Efficacy of the BNT162b2 mRNA COVID-19 Vaccine. N. Engl. J. Med..

[5] Armocida B., Formenti B., Ussai S., Palestra F., Missoni E. (2020). The Italian health system and the COVID-19 challenge. Lancet Public Health.

[6] Weinreich D.M., Sivapalasingam S., Norton T., Ali S., Gao H., Bhore R., Xiao J., Hooper A.T., Hamilton J.D., Musser B.J. (2021). REGEN-COV Antibody Cocktail Clinical Outcomes Study in COVID-19 Outpatients. medRxiv.

[7] COVID-19—Situazione in Italia. https://www.salute.gov.it/portale/nuovocoronavirus/dettaglioContenutiNuovoCoronavirus.jsp?area=nuovoCoronavirus&id=5351&lingua=italiano&menu=vuoto.

[8] CovdiStat INFN—Dati dell’ISS. https://covid19.infn.it/iss/.

[9] Dougan M., Nirula A., Azizad M., Mocherla B., Gottlieb R.L., Chen P., Hebert C., Perry R., Boscia J., Heller B. (2021). Bamlanivimab plus Etesevimab in Mild or Moderate COVID-19. N. Engl. J. Med..

[10] Gupta A., Gonzalez-Rojas Y., Juarez E., Casal M.C., Moya J., Falci D.R., Sarkis E., Solis J., Zheng H., Scott N. (2021). Early COVID-19 Treatment With SARS-CoV-2 Neutralizing Antibody Sotrovimab. medRxiv.

[11] US Demand for COVID-19 Antibody Treatments Rising Fast—PMLiVE. https://www.pmlive.com/pharma_news/us_demand_for_COVID-19_antibody_treatments_rising_fast_1376021.

[12] Kohli M., Maschio M., Becker D., Weinstein M.C. (2021). The potential public health and economic value of a hypothetical COVID-19 vaccine in the United States: Use of cost-effectiveness modeling to inform vaccination prioritization. Vaccine.

